# Antifungal resistance and poor clinical outcome rates of *Candida auris* in mainland China: A systematic review and meta-analysis

**DOI:** 10.3389/fmicb.2026.1888683

**Published:** 2026-08-31

**Authors:** Tao Xu, Rui Wang, Yufang Gao, Lei Yang

**Affiliations:** 1 Department of Clinical Laboratory, Xianyang Central Hospital, Xianyang, Shaanxi, China; 2 Department of Reproductive Medicine, Xianyang Central Hospital, Xianyang, Shaanxi, China

**Keywords:** antifungal drug resistance, *Candida auris*, *Candidozyma auris*, clinical deterioration, meta-analysis, mortality

## Abstract

**Objective:**

This study systematically evaluated the antifungal resistance profile of *Candida auris* (*C. auris*) to five antifungal agents in mainland China, and analyzed clinical outcomes in a combined cohort of patients with *C. auris* colonization or infection. It aims to provide evidence-based references for clinical antifungal management and nosocomial infection prevention and control.

**Methods:**

Six databases, including PubMed, Web of Science, and Embase, were systematically searched to identify observational studies reporting antifungal susceptibility of *C. auris* isolates and clinical outcomes of corresponding patients in mainland China. Meta-analysis was adopted to pool the resistance rates and multidrug resistance (MDR) rate of five antifungal agents. Meanwhile, we summarized the overall rate of poor prognosis, in-hospital mortality rate, and rate of clinical deterioration at discharge for patients with detected *C. auris*. Funnel plots and Egger’s test were used to assess publication bias.

**Results:**

*C. auris* in mainland China exhibited extremely high resistance to fluconazole, with a pooled resistance rate of 98% (95%CI: 93%–100%). Echinocandins maintained favorable overall susceptibility. The resistance rates of caspofungin, micafungin, and anidulafungin were 3% (95%CI: 0%–11%), 2% (95%CI: 0%–4%), and 1% (95%CI: 0%–3%), respectively. The resistance rate of *C. auris* to amphotericin B was 34% (95%CI: 12%–61%). The pooled MDR rate was 60% (95% CI: 16%–97%). The overall poor prognosis rate of patients with *C. auris* isolation was 43% (95%CI: 23%–64%). The in-hospital mortality rate was 24% (95% CI: 12%–38%), while the rate of clinical deterioration at discharge was 23% (95% CI: 5%–46%). No significant publication bias was observed in this study.

**Conclusion:**

Fluconazole is not an appropriate choice for treating *C. auris* strains in mainland China, while echinocandins should be the primary treatment option. Amphotericin B should be administered cautiously based on antifungal susceptibility testing. The pooled MDR rate was relatively high. Patients with *C. auris* colonization or infection exhibit variable clinical outcomes. Clinical practice should prioritize routine laboratory antifungal susceptibility surveillance, standardized hospital infection control measures, and targeted interventions to improve patient clinical outcomes.

## 1 Introduction

*Candida auris* (*C. auris*), recently taxonomically reclassified as *Candidozyma auris* (Liu F. et al., 2024), is an emerging multidrug-resistant fungal pathogen associated with a high mortality rate (Forsberg et al., 2019). This fungal pathogen was first isolated and identified from the ear canal secretions of a female patient in Japan in 2009 (Satoh et al., 2009). It has since rapidly spread across Asia, Europe, Africa, North America, and South America (Achilonu et al., 2025; Bing et al., 2026). Due to its strong transmissibility and clinical resistance, it has significantly increased the medical and economic burden in various countries, posing a major threat to global public health. In 2019, the Centers for Disease Control and Prevention of the United States classified it as a pathogen posing an urgent public health threat (Centers for Disease Control and Prevention, 2019). The fungus readily colonizes human skin for extended periods, survives stably on environmental surfaces, and exhibits extensive resistance to antifungal agents. These features make control of *C. auris* transmission difficult, and the prognosis for patients is often poor (Du et al., 2022; Ji et al., 2026; Lionakis and Chowdhary, 2024). Accordingly, the World Health Organization classified it as a critical priority fungal pathogen in 2022 (World Health Organization [WHO], 2022). The first *C. auris* case in mainland China was identified in 2018 (Wang et al., 2018). Since then, clinical reports of this pathogen have accumulated continuously. Its multidrug-resistant profile and poor clinical prognosis bring great challenges to nosocomial infection control and clinical care.

In clinical practice, fluconazole, caspofungin, micafungin, anidulafungin, and amphotericin B are conventional antifungal agents for fungal infections (Perfect, 2017). Although these drugs have been applied in the clinical treatment of *C. auris* infection, relevant studies on the antifungal resistance of *C. auris* in mainland China are mostly limited to case reports and small-sample analyses (Bing et al., 2022; Yang et al., 2025), failing to reflect the overall epidemiological characteristics of drug resistance. Previous surveillance data from mainland China revealed extremely high fluconazole resistance among *C. auris* isolates, constituting a major clinical antifungal challenge (Bing et al., 2025). A moderate proportion of isolates display amphotericin B resistance, while resistance to echinocandins remains relatively low. It is noteworthy that there is currently a lack of systematic summary data on the resistance rates of *C. auris* to fluconazole, amphotericin B, and echinocandin drugs (caspofungin, micafungin, anidulafungin) in mainland China, making it difficult to obtain reliable antifungal susceptibility references and establish targeted hospital infection control strategies for the clinical treatment of such *C. auris* infections.

Current evidence describing patient prognosis following *C. auris* isolation within mainland China shows notable inconsistency (Wang et al., 2025; Zhang H. et al., 2025). Divergent rates of adverse clinical events reported across studies arise from disparities in sample scale, differing severity of underlying chronic illnesses, and variable study architectures. Most relevant regional clinical records derive from single-center retrospective analyses with small sample sizes, which weakens the reliability of individual results and creates contradictory observational conclusions. In addition, few studies have comprehensively evaluated the in-hospital mortality rate and the rate of clinical deterioration at discharge for patients with *C. auris* isolation, which limits the comprehensive understanding of clinical outcomes and the formulation of intervention strategies for such patients.

To resolve discrepancies among published single-center studies, we performed a systematic review and meta-analysis to integrate those studies focusing on *C. auris* in mainland China, and systematically analyze the resistance rates of five core antifungal drugs, including fluconazole, caspofungin, micafungin, anidulafungin, and amphotericin B. Meanwhile, this study performed a pooled analysis of poor prognostic outcomes among patients with *C. auris* colonization or infection. We further stratified poor prognoses into in-hospital mortality rate and the rate of clinical deterioration at discharge, and conducted separate subgroup analyses. This study aims to clarify the current status of antifungal resistance, in-hospital mortality, and clinical deterioration at discharge among patients with *C. auris* isolation in mainland China, thereby providing evidence-based references for the rational clinical use of antifungal agents and reducing adverse clinical outcomes.

## 2 Materials and methods

### 2.1 Literature search

This study was conducted and written in accordance with the Preferred Reporting Items for Systematic Reviews and Meta-Analyses (PRISMA) guidelines (Moher et al., 2009). The research protocol was prospectively registered on the PROSPERO platform with the registration number CRD420261363661, and the detailed design can be accessed via the official link^1^.

We searched three English databases: PubMed, Web of Science, and Embase. Also three Chinese databases: CNKI, Wanfang Medical Network, and VIP Chinese Journal Database. For PubMed, we used “*Candida auris*” or “*Candidozyma auris*” as search terms, limited to titles and abstracts, and restricted the results to affiliations with institutions in China. The other databases were searched in a similar way. The retrieval period started from when each database was first established up to April 8, 2026. The goal was to collect as many clinical studies related to *C. auris* in mainland China as possible, and to pull out indicators on drug resistance and prognosis. We also manually checked the reference lists of the included articles to make sure we didn’t miss anything, trying to keep the results as complete as we could.

### 2.2 Inclusion and exclusion criteria

#### 2.2.1 Inclusion criteria

(1) studies conducted in mainland China; (2) study subjects were individuals positive for *C. auris*, with no fewer than five enrolled patients or isolates; (3) articles that fully reported antifungal susceptibility data of *C. auris* or adverse clinical outcome data of patients for the same type of specimens from the same patient, only the first detection result was included.

#### 2.2.2 Exclusion criteria

(1) Reviews, case reports with sample size less than 5 cases, clinical experience summaries, drug resistance mechanism studies, detection methodology studies and literatures on nosocomial infection prevention and control; (2) animal experimental studies; (3) duplicate published data, repeated detection results of the same type of specimens from the same patient, and studies conducted outside Chinese mainland were excluded; (4) literatures lacking core indicators of drug resistance or prognosis, from which original data could not be obtained even by contacting the authors.

### 2.3 Data extraction and quality evaluation

#### 2.3.1 Data extraction

Antifungal resistance of *C. auris* was defined according to the clinical breakpoints published by the United States Centers for Disease Control and Prevention^2^. The thresholds were as follows: fluconazole ≥32 μg/mL, caspofungin ≥2 μg/mL, micafungin ≥4 μg/mL, anidulafungin ≥4 μg/mL, and amphotericin B ≥2 μg/mL. Based on the above breakpoints for antifungal susceptibility, multidrug resistance (MDR) was defined as acquired non-susceptibility to no less than two of the three tested antifungal classes. Only studies providing complete susceptibility results for all three drug categories were included for this analysis. Poor prognosis in this study was defined as in-hospital death or clinical deterioration at discharge. This deterioration was defined as meeting any of the following criteria: (a) Progressive worsening of relevant clinical manifestations relative to admission (including but not limited to fever, respiratory status, and circulatory function), or unknown outcome data; (b) discharge assessment using established functional scales, such as the Glasgow Coma Scale or the modified Rankin Scale, indicated that the surviving patient remained in a critically ill or end-stage condition; (c) continued clinical deterioration prompting the attending physician to withdraw active treatment; (d) clinical instability necessitating transfer to another facility for escalated care; or (e) failure to show clinical improvement, with the family requesting early discharge against medical advice.

EndNote X9 software was used for literature management. Two researchers (Rui Wang, Yufang Gao) independently screened the titles, abstracts, and full texts of the articles strictly according to the inclusion and exclusion criteria. Subsequently, the two researchers independently extracted clinical data from included literatures, including first author, publication year, research region, research period, age of study subjects, underlying diseases, combined infections, antimicrobial susceptibility testing instruments, number of drug-resistant strains of five antifungal agents (fluconazole, caspofungin, micafungin, anidulafungin, amphotericin B), drug resistance genes, and adverse outcome indicators of patients including in-hospital mortality cases and post-discharge deterioration cases. Any disagreement in literature screening or data extraction was resolved through negotiation with a senior third researcher (Lei Yang). Where several reports appeared to stem from a shared source population, we kept the single study with the biggest enrollment and excluded the others, so as not to count any patient more than once. That said, constrained by what the primary sources provided, we cannot rule out some residual overlap among the included studies.

#### 2.3.2 Quality evaluation

The same two researchers (Rui Wang, Yufang Gao) independently evaluated the methodological quality of included studies using the Agency for Healthcare Research and Quality (AHRQ) scale (Rostom et al., 2004). Disagreements were resolved through discussion with the third researcher (Lei Yang). The AHRQ scale consists of 11 items, with three options of “Yes”, “No“, and “Unclear” for each item; one point is assigned for “Yes”, and zero points for “No” or “Unclear”, with a total score ranging from 0 to 11 points. Studies were divided into three grades according to the total score: high quality (≥8 points), moderate quality (4–7 points), and low quality (≤3 points).

### 2.4 Statistical analysis

All patients presenting with *C. auris* colonization or infection as documented in eligible studies formed a combined cohort and were included in the quantitative synthesis. The antifungal resistance indicators analyzed in this study included the resistance rates of *C. auris* to fluconazole, caspofungin, micafungin, anidulafungin, and amphotericin B. In addition, we pooled relevant data to calculate the MDR rate. The poor prognostic indicators for patients included the overall poor prognosis rate, as well as the subdivided in-hospital mortality rate and the rate of clinical deterioration at discharge. All statistical analyses were performed using R software (version 4.2.0; R Foundation for Statistical Computing, Vienna, Austria) with the meta package (version 8.3-0). In this study, the pooled positive rates were calculated using the double arcsine transformation method (Barker et al., 2021) and 95% confidence intervals (CIs) were provided. Forest plots were used to display the effect size of each study and the pooled results. Heterogeneity among included studies was assessed by the *I*^2^ statistic and the *P* value of Cochran’s *Q* test (Higgins et al., 2023). When *I*^2^ ≤ 50% and *P* > 0.1, it was determined as low heterogeneity. The fixed-effect model (inverse variance method) was used to pool the data, and 95% CIs were calculated by the classical method. If *I*^2^ > 50% or *P* < 0.1, the heterogeneity was considered substantial. In those cases, we used a random-effects model with restricted maximum likelihood (REML) estimation (Langan et al., 2019), adjusting the 95% CI using the Hartung–Knapp–Sidik–Jonkman (HKSJ) method (Hartung and Knapp, 2001). To explore the sources of heterogeneity, we carried out subgroup analyses based on antifungal susceptibility testing methods, geographic region, and mutations in the ERG11 and FKS1 genes. The funnel plots were used to visually assess publication bias. If the graph was roughly symmetrical, we considered that the risk of publication bias was relatively low. Furthermore, Egger’s linear regression test was adopted to assess publication bias for each indicator when the number of included studies was 10 or more. The significance level was set at 0.1.

## 3 Results

### 3.1 Literature search and quality evaluation of included studies

**FIGURE 1:**
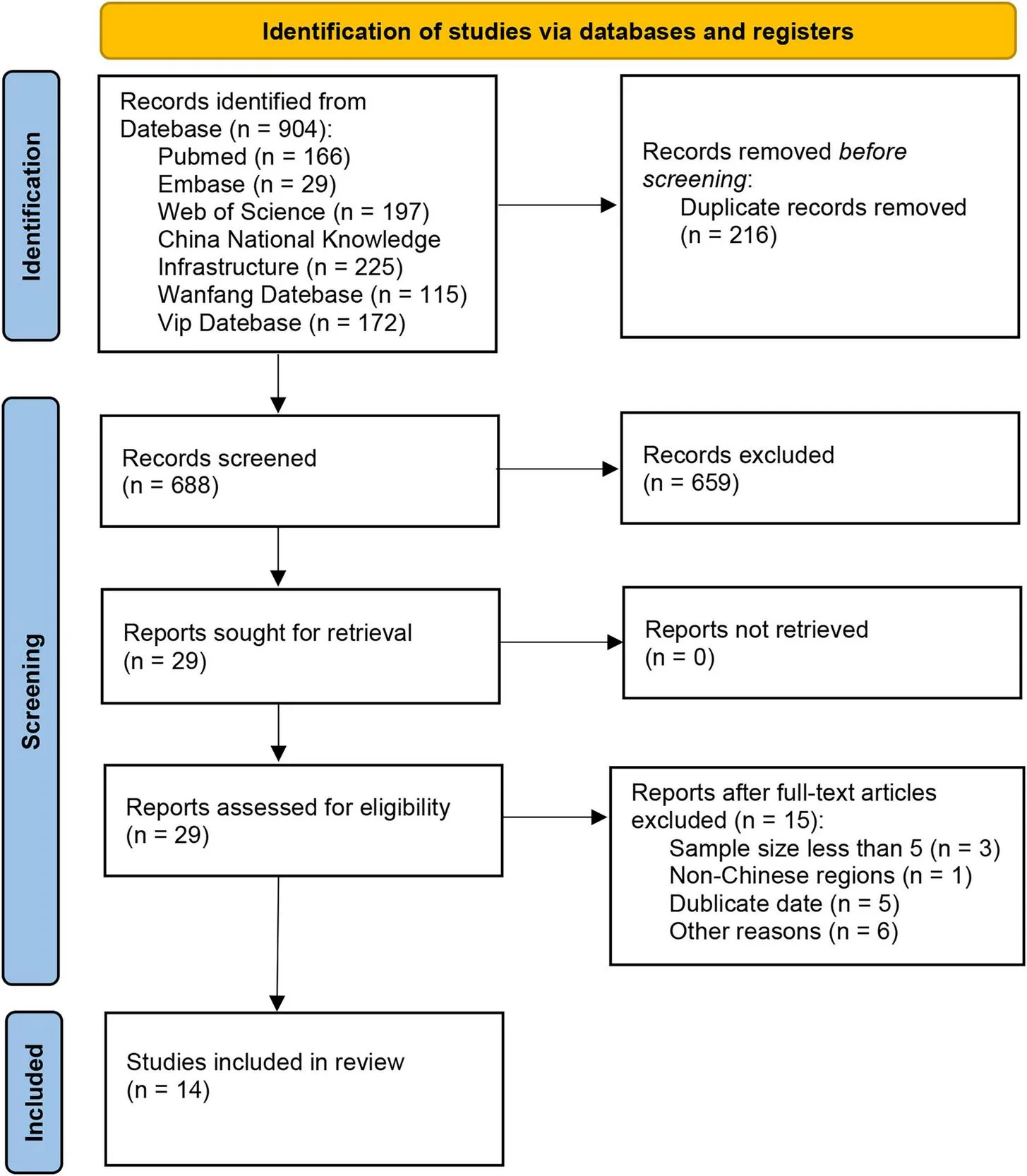
Flowchart of the included studies selection process.

**FIGURE 2:**
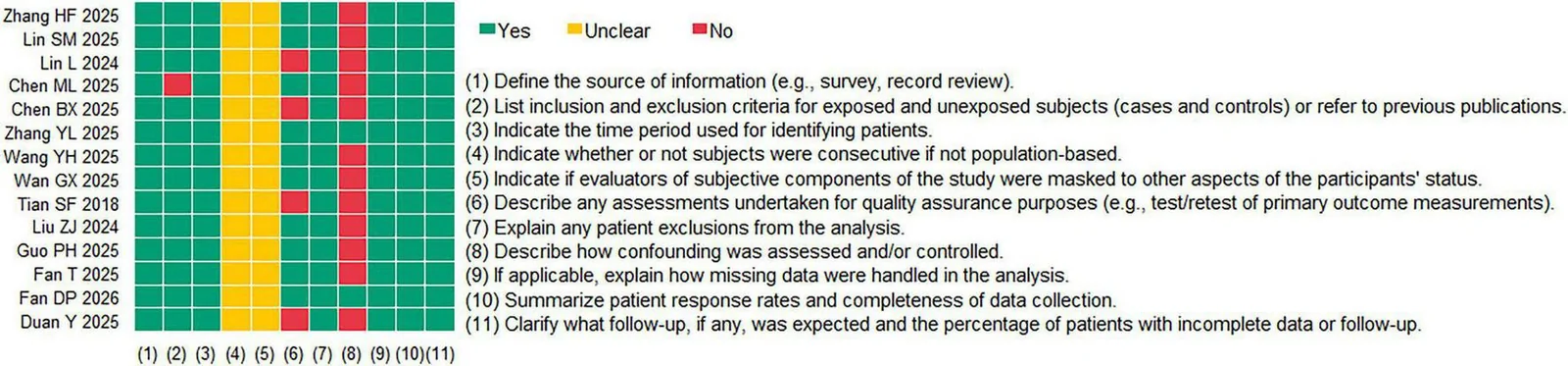
Quality assessment results of included studies.

A total of 904 relevant articles were retrieved, including 392 from English databases and 512 from Chinese databases. After removing 216 duplicate records via EndNote X9, 202 irrelevant records were excluded by reading titles, abstracts, and full texts. Ultimately, 14 observational studies were included for meta-analysis (Chen and Feng, 2025; Chen et al., 2025; Duan et al., 2025; Fan et al., 2025, 2026; Guo et al., 2025; Lin et al., 2024, 2025; Liu F. et al., 2024; Tian et al., 2018; Wan et al., 2025; Wang et al., 2025; Zhang H. et al., 2025; Zhang Y. et al., 2025), and the literature screening process is shown in Figure 1. Quality evaluation of included literature was conducted according to the AHRQ criteria, with all scores no less than 7 points, including 9 high-quality studies (Fan et al., 2025, 2026; Guo et al., 2025; Lin et al., 2025; Liu F. et al., 2024; Wan et al., 2025; Wang et al., 2025; Zhang H. et al., 2025; Zhang Y. et al., 2025) and 5 moderate-quality studies (Chen and Feng, 2025; Chen et al., 2025; Duan et al., 2025; Lin et al., 2024; Tian et al., 2018). The quality evaluation results are presented in Figure 2.

### 3.2 Basic characteristics of the included literatures

The included studies were published from 2018 to 2026, involving 4 studies in northern China (Chen et al., 2025; Liu F. et al., 2024; Tian et al., 2018; Zhang Y. et al., 2025) and 10 studies in southern China (Chen and Feng, 2025; Duan et al., 2025; Fan et al., 2025, 2026; Guo et al., 2025; Lin et al., 2024, 2025; Wan et al., 2025; Wang et al., 2025; Zhang H. et al., 2025). A total of 582 patients with *C. auris* colonization or infection were included, most of whom were admitted to the intensive care unit. The baseline characteristics of the included studies are shown in Table 1. Among them, 14 studies reported fluconazole resistance (Chen and Feng, 2025; Chen et al., 2025; Duan et al., 2025; Fan et al., 2025, 2026; Guo et al., 2025; Lin et al., 2024, 2025; Liu F. et al., 2024; Tian et al., 2018; Wan et al., 2025; Wang et al., 2025; Zhang H. et al., 2025; Zhang Y. et al., 2025); 11 reported caspofungin resistance (Chen et al., 2025; Fan et al., 2025, 2026; Guo et al., 2025; Lin et al., 2024; Liu F. et al., 2024; Tian et al., 2018; Wan et al., 2025; Wang et al., 2025; Zhang H. et al., 2025; Zhang Y. et al., 2025); 9 reported micafungin resistance (Chen et al., 2025; Fan et al., 2026; Guo et al., 2025; Lin et al., 2024; Liu F. et al., 2024; Tian et al., 2018; Wan et al., 2025; Wang et al., 2025; Zhang Y. et al., 2025); 9 reported anidulafungin resistance (Chen et al., 2025; Fan et al., 2026; Guo et al., 2025; Lin et al., 2024; Liu F. et al., 2024; Tian et al., 2018; Wan et al., 2025; Wang et al., 2025; Zhang Y. et al., 2025); 14 reported amphotericin B resistance (Chen and Feng, 2025; Chen et al., 2025; Duan et al., 2025; Fan et al., 2025, 2026; Guo et al., 2025; Lin et al., 2024, 2025; Liu F. et al., 2024; Tian et al., 2018; Wan et al., 2025; Wang et al., 2025; Zhang H. et al., 2025; Zhang Y. et al., 2025). Additionally, 12 reported adverse prognostic outcomes (Chen and Feng, 2025; Chen et al., 2025; Duan et al., 2025; Fan et al., 2025, 2026; Guo et al., 2025; Lin et al., 2024, 2025; Liu F. et al., 2024; Wang et al., 2025; Zhang H. et al., 2025; Zhang Y. et al., 2025), including 11 for mortality (Chen and Feng, 2025; Chen et al., 2025; Duan et al., 2025; Fan et al., 2025, 2026; Guo et al., 2025; Lin et al., 2024; Liu F. et al., 2024; Wang et al., 2025; Zhang H. et al., 2025; Zhang Y. et al., 2025) and 8 for clinical deterioration (Chen et al., 2025; Duan et al., 2025; Fan et al., 2026; Guo et al., 2025; Lin et al., 2024; Liu F. et al., 2024; Wang et al., 2025; Zhang Y. et al., 2025).

**TABLE 1:** Basic characteristics of the included studies.

Study	Year	Region	Number of patients (*n*)	Male/Female (*n*)	Infection/ Colonization (*n*)	Age (years)	Antifungal susceptibility testing instrument	Clade classification (*n*)	*ERG11* mutation
Zhang H. et al., 2025	2025	Guangdong	12	10/2	2/10	21–92	VITEK-2	Unknow	Unknow
Lin et al., 2025	2025	Guangdong	103	72/31	103/0	≥18	ATB Fungus 3	Unknow	Unknow
Lin et al., 2024	2024	Guangdong	10	8/2	Unknow	38–77	AutoMic-i600	Unknow	Unknow
Chen et al., 2025	2025	Beijing	9	3/6	5/4	41–91	Unknow	Unknow	Unknow
Chen and Feng, 2025	2025	Guangdong	178	125/53	Unknow	>60 (*n* = 153)	ATB Fungus 3	Unknow	Unknow
Chen and Feng, 2025	2025	Beijing	34	20/14	Unknow	34–96	Sensititre YeastOne	Clade I (35)	All fluconazole-resistant isolates
Wang et al., 2025	2025	Guangdong	7	Unknow	Unknow	64–90	Unknow	Clade I (3); Clade III (4)	All fluconazole-resistant isolates
Wan et al., 2025	2025	Guangdong	37	24/13	Unknow	41–97	Sensititre YeastOne	Clade I (29); Clade III (10)	All fluconazole-resistant isolates
Tian et al., 2018	2018	Jilin	15	8/7	Unknow	49–86	Sensititre YeastOne	Unknow	Unknow
Liu F. et al., 2024	2024	Beijing	17	14/3	13/4	26–86	Sensititre YeastOne	Clade I (17)	All fluconazole-resistant isolates
Guo et al., 2025	2025	Guangdong	108	74/34	Unknow	22–83	Sensititre YeastOne	Clade I (15); Clade III (93)	All fluconazole-resistant isolates
Fan et al., 2025	2025	Guangdong	31	22/9	31/0	≥18	ATB Fungus 3	Unknow	Unknow
Fan et al., 2026	2026	Shanghai	10	7/3	0/10	56–79	Sensititre YeastOne	Clade I (1); Clade III (9)	Unknow
Fan et al., 2026	2025	Guangdong	11	10/1	11/0	42–93	ATB Fungus 3	Unknow	Unknow
**Study**	**Coinfection (*n*)**			**Comorbidities (*n*)**			**Invasive procedures (n)**		
Zhang H. et al., 2025	Pulmonary infection (11), urinary tract infection (6), bloodstream infection (5), others			Hypertension (9), diabetes mellitus (3), chronic obstructive pulmonary disease (2)			Craniocerebral surgery (5), cardiac surgery (1), thoracic spine surgery (1)		
Lin et al., 2025	Pneumonia (98), sepsis (12), other fungal infections (22)			Hypertension (57), diabetes mellitus (30), respiratory failure (13), heart failure (17)			Surgical intervention (34)		
Lin et al., 2024	Pulmonary infection (4), pneumonia (2), bloodstream infection (3), others (6)			Diabetes mellitus (1), necrotizing fasciitis (2), neoplasm (2)			Bronchoalveolar lavage (2), catheter (1), venous catheterization (1)		
Chen et al., 2025	Pneumonia (5), systemic infection (2), septic shock (1)			Hypertension (1), diabetes mellitus (2), respiratory failure (3), neoplasm (2)			Abdominal drainage (1)		
Chen and Feng, 2025	Other pathogen infections (41)			Diabetes mellitus (33), urinary system diseases (108), malignant tumor (12)			Tracheal intubation (108), drainage tube (8), dialysis (8)		
Chen and Feng, 2025	Severe pneumonia (22)			Hypertension (26), diabetes mellitus (15), chronic obstructive pulmonary disease (7), heart failure (6), others			Catheter/hemodialysis/mechanical ventilation (31)		
Wang et al., 2025	Unknow			All patients had severe underlying diseases			Central venous catheter/peripherally inserted central catheter (7)		
Wan et al., 2025	Unknow			Unknow			Central venous catheter (3)		
Tian et al., 2018	Pneumonia (10)			Diabetes mellitus (7), central nervous system(10), lung dysfunction(9), malignancy(3), others			Urinary catheter (Foley) (15), mechanical ventilation (14), trachea intubation (10), central venous line (8), others		
Liu F. et al., 2024	Unknow			Critically ill (14)			Central venous catheter(4)		
Guo et al., 2025	Unknow			Unknow			Indwelling urinary catheters (98), mechanical ventilation (79)		
Fan et al., 2025	Pulmonary infections (15), urinary tract infections (7), bloodstream infections (3), others(6)			Hypertension (17), immunosuppressive status (10), diabetes mellitus (8), others			Indwelling medical devices (20)		
Fan et al., 2026	Urinary tract infections (7), bloodstream infection (2)			Hypertension (3), diabetes mellitus (6)			Indwelling urinary catheters (10)		
Fan et al., 2026	Pneumonia (8)			Hypertension (6), diabetes mellitus (4), cerebral infarction or haemorrhage (5), others			Peripherally inserted central venous catheter (10), urinary catheters (9), mechanical ventilation (1)		
**Study**	**Specimens (*n*)**			**Outbreak characteristics**			**Infection control measures**		
Zhang H. et al., 2025	Urine (10), sputum (3), deep venous catheter (1), tissue biopsy specimen (1), wound swab (1)			Sporadic cases			Poor staff awareness of *C. auris*; irregular indwelling catheter care		
Lin et al., 2025	Urine (76), respiratory specimens (14), blood (9), others (14)			Unknow			Unknow		
Lin et al., 2024	Aspirated sputum (2), bronchoalveolar lavage fluid (2), venous blood (2), others (4)			Unknow			Unknow		
Chen et al., 2025	Urine (5), sputum (3), abdominal drainage fluid (1)			Unknow			Patient isolation; environmental microbial surveillance; environmental disinfection; hand hygiene; other interventions		
Chen and Feng, 2025	Urine (108), respiratory specimens (31), traumatic secretions (18), blood (8), others (13)			Unknow			Unknow		
Zhang Y. et al., 2025	Groin, axilla, urine, bronchoalveolar lavage fluid, others			Ward outbreaks; sporadic cases			Unknow		
Wang et al., 2025	Blood (1), urine (2), sputum (2), bronchoalveolar lavage fluid (2)			Unknow			Environmental microbial surveillance		
Wan et al., 2025	Urine (11), sputum (10), blood (5), central venous catheter (3), ascites fluid (1), cerebrospinal fluid (1), others			Unknow			Unknow		
Tian et al., 2018	Urine (10), blood (1), catheter (2), sputum (1), drainage (1)			Sporadic cases			Unknow		
Liu F. et al., 2024	Urine (12), blood (7), central venous catheter (4), bronchoalveolar lavage fluid (1)			Ward outbreaks			Environmental microbial surveillance; environmental disinfection; personal protective equipment; hand hygiene; reprocessed medical device management		
Guo et al., 2025	Sputum/bronchoalveolar lavage fluid (36), urine (34), blood (10), drainage fluid (9), others (15)			Unknow			Unknow		
Fan et al., 2025	Unknow			Unknow			Unknow		
Fan et al., 2026	Urine (10)			Sporadic cases			Weekly multi-site patient screening; environmental and healthcare worker hand sampling		
Duan et al., 2025	Blood (11)			Unknow			Patient isolation; environmental disinfection; targeted nursing care		

Zhang Y. et al. (2025) reported that all 35 isolates belonged to the South Asian clade (Clade I) and carried the *ERG11* (Y132F) mutation. Three of these isolates were resistant to caspofungin with different *FKS1* gene mutation sites, including F635I, R1354H, and W1471L. Wang et al. (2025) documented that the 7 isolates comprised 4 strains belonging to the South African clade (Clade III) and 3 to the South Asian clade (Clade I). All isolates carried *ERG11* gene mutations. Five harbored the V125A and F126L mutations. The remaining two lacked missense mutations at positions 125 and 126; one of these two carried the Y132F mutation instead. Wan et al. (2025) found that of the 39 isolates, 29 were Clade I strains and 10 were Clade III strains. All isolates carried *ERG11* gene mutations, with the K143R mutation in Clade I strains and the F126L mutation in Clade III strains. Liu Z. et al. (2024) reported that all 17 isolates belonged to Clade I and harbored the *ERG11* (Y132F) mutation. Of the 3 caspofungin-resistant isolates, 2 were found to carry the *FKS1* (S639F) mutation, whereas the remaining one was not subjected to *FKS1* genetic testing. Guo et al. (2025) identified 108 isolates, including 93 South African clade (Clade III) strains and 15 South Asian clade (Clade I) strains. All isolates carried *ERG11* gene mutations: Clade III strains harbored the V125A mutation, while Clade I strains had the Y132F mutation. In the present study, all 8 caspofungin-resistant isolates were classified as Clade III and carried *FKS1* mutations, among which 5 harbored the S639Y mutation, 2 carried the S639F mutation, and 1 had the W691L mutation. Fan et al. (2026) identified a single caspofungin-resistant isolate harboring the S639F mutation in the *FKS1* gene.

### 3.3 Meta-analysis of antifungal resistance rates in *C. auris*

#### 3.3.1 Pooled fluconazole resistance rate

**FIGURE 3:**
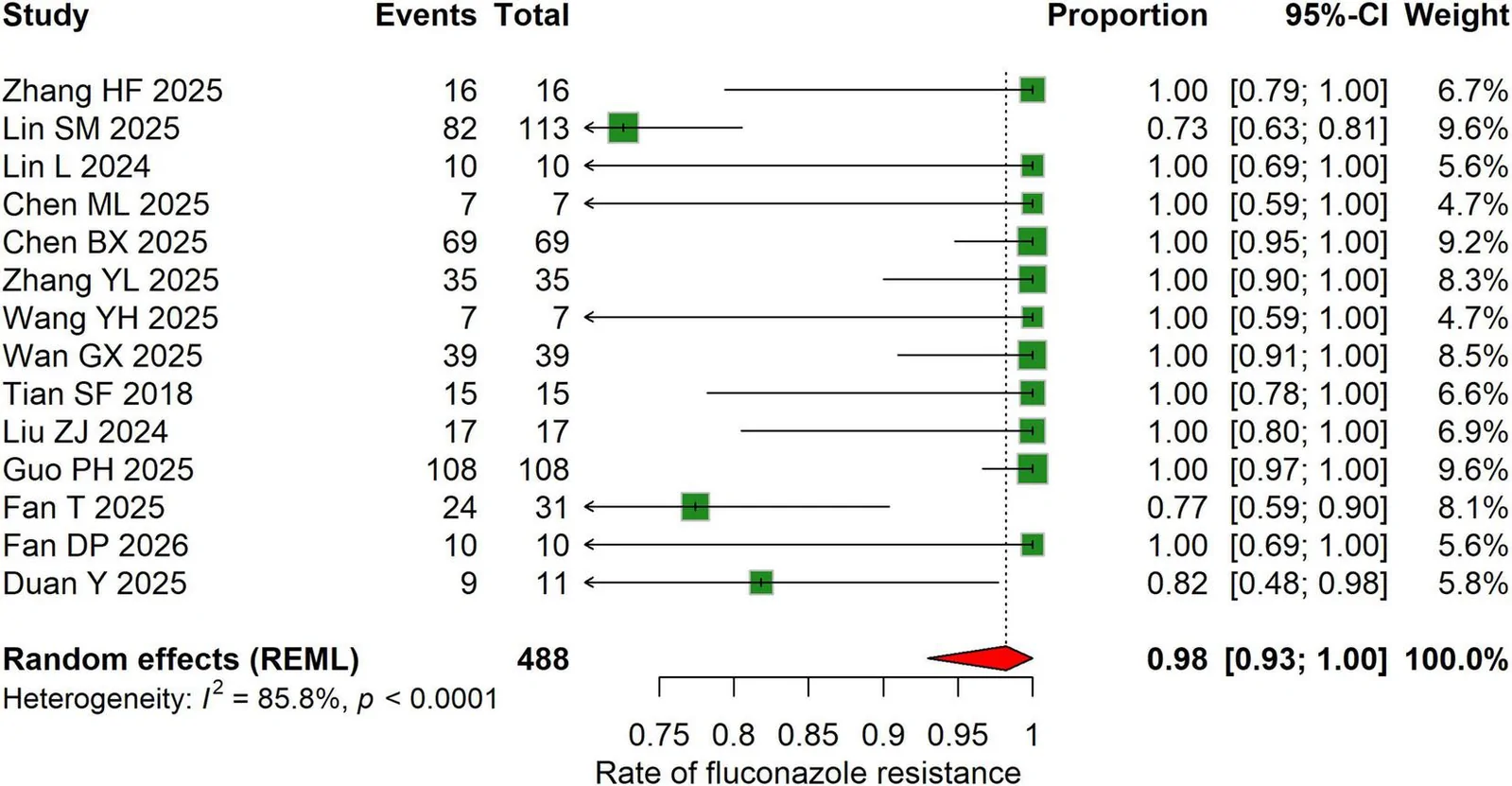
Forest plot of fluconazole resistance rate in *C. auris* isolates.

A total of 488 *C. auris* strains were tested for susceptibility to fluconazole, among which 448 were drug-resistant strains. The random-effect model was adopted for pooled analysis, and the results showed that the pooled fluconazole resistance rate was 98% (95% CI: 93%–100%), with significant heterogeneity across studies (*I*^2^ = 85.8%, *P* < 0.0001) (Figure 3).

#### 3.3.2 Pooled caspofungin resistance rate

**FIGURE 4:**
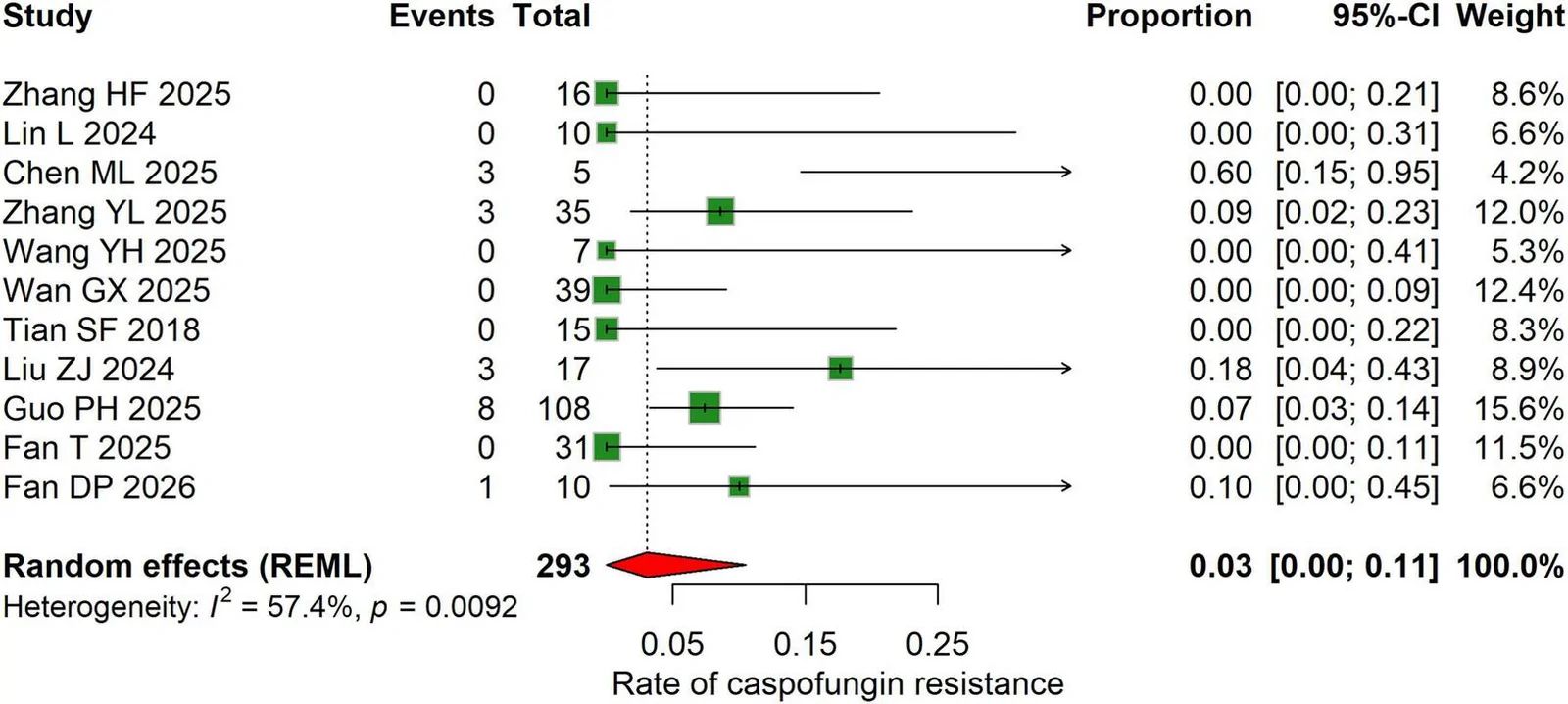
Forest plot of caspofungin resistance rate in *C. auris* isolates.

Antifungal susceptibility testing of caspofungin was performed on 293 strains, including 18 resistant strains. The random-effect model was used for pooled analysis, and the overall caspofungin resistance rate was 3% (95% CI: 0%–11%), with statistically significant heterogeneity among included studies (*I*^2^ = 57.4%, *P* = 0.0092) (Figure 4).

#### 3.3.3 Pooled micafungin resistance rate

**FIGURE 5:**
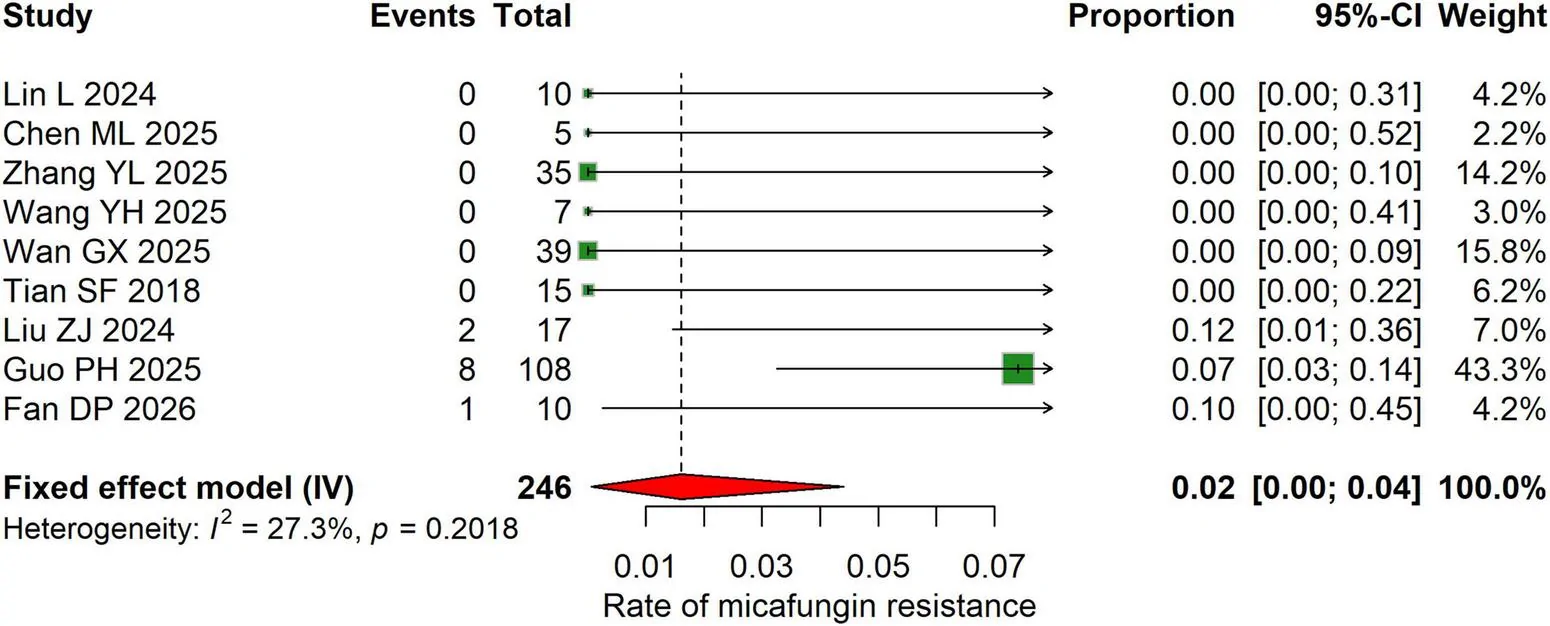
Forest plot of micafungin resistance rate in *C. auris* isolates.

Antifungal susceptibility testing of micafungin was conducted on 246 strains, and 11 resistant strains were identified. Pooled analysis via the fixed-effect model showed that the pooled micafungin resistance rate was 2% (95% CI: 0%–4%), with low heterogeneity across studies (*I*^2^ = 27.3%, *P* = 0.2018) (Figure 5).

#### 3.3.4 Pooled anidulafungin resistance rate

**FIGURE 6:**
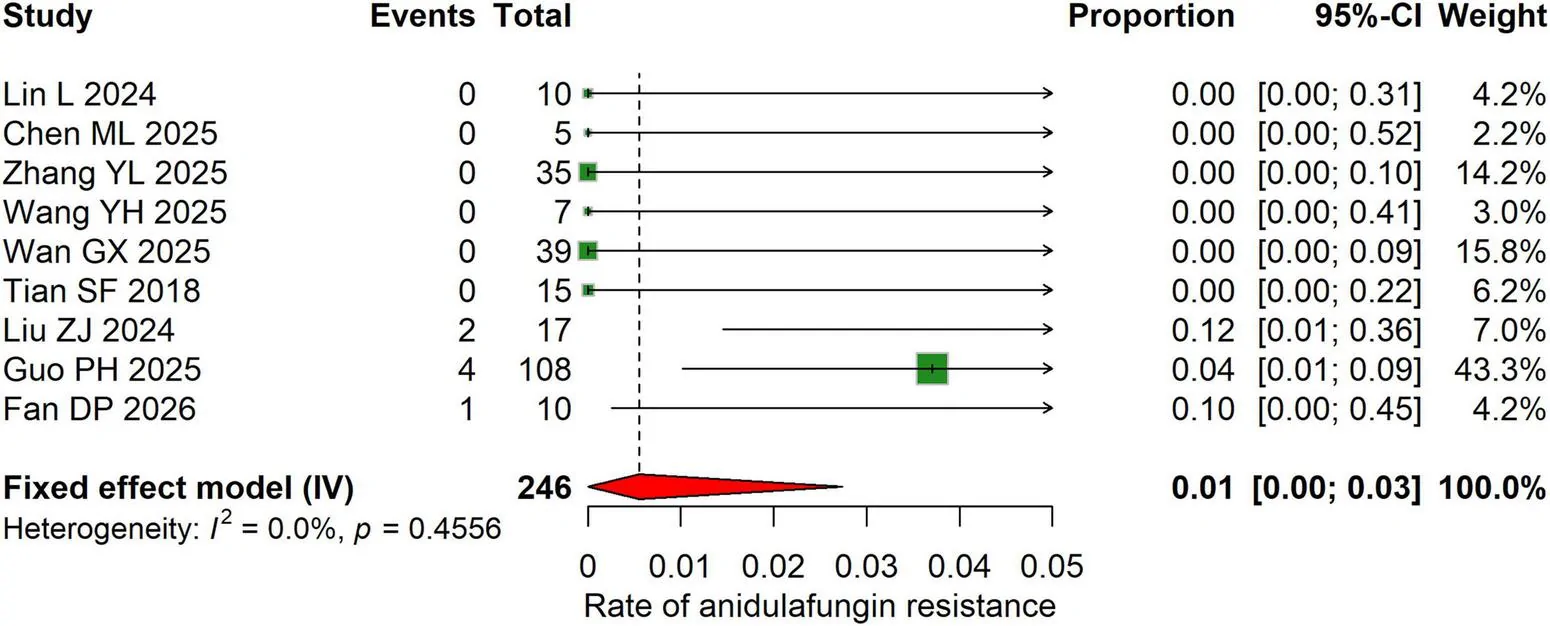
Forest plot of anidulafungin resistance rate in *C. auris* isolates.

Among 246 strains completed with anidulafungin susceptibility testing, 7 were resistant strains. Comprehensive analysis using the fixed-effect model revealed that the overall anidulafungin resistance rate was 1% (95% CI: 0%–3%), with no statistically significant heterogeneity among included studies (*I*^2^ = 0%, *P* = 0.4556) (Figure 6).

#### 3.3.5 Pooled amphotericin B resistance rate

**FIGURE 7:**
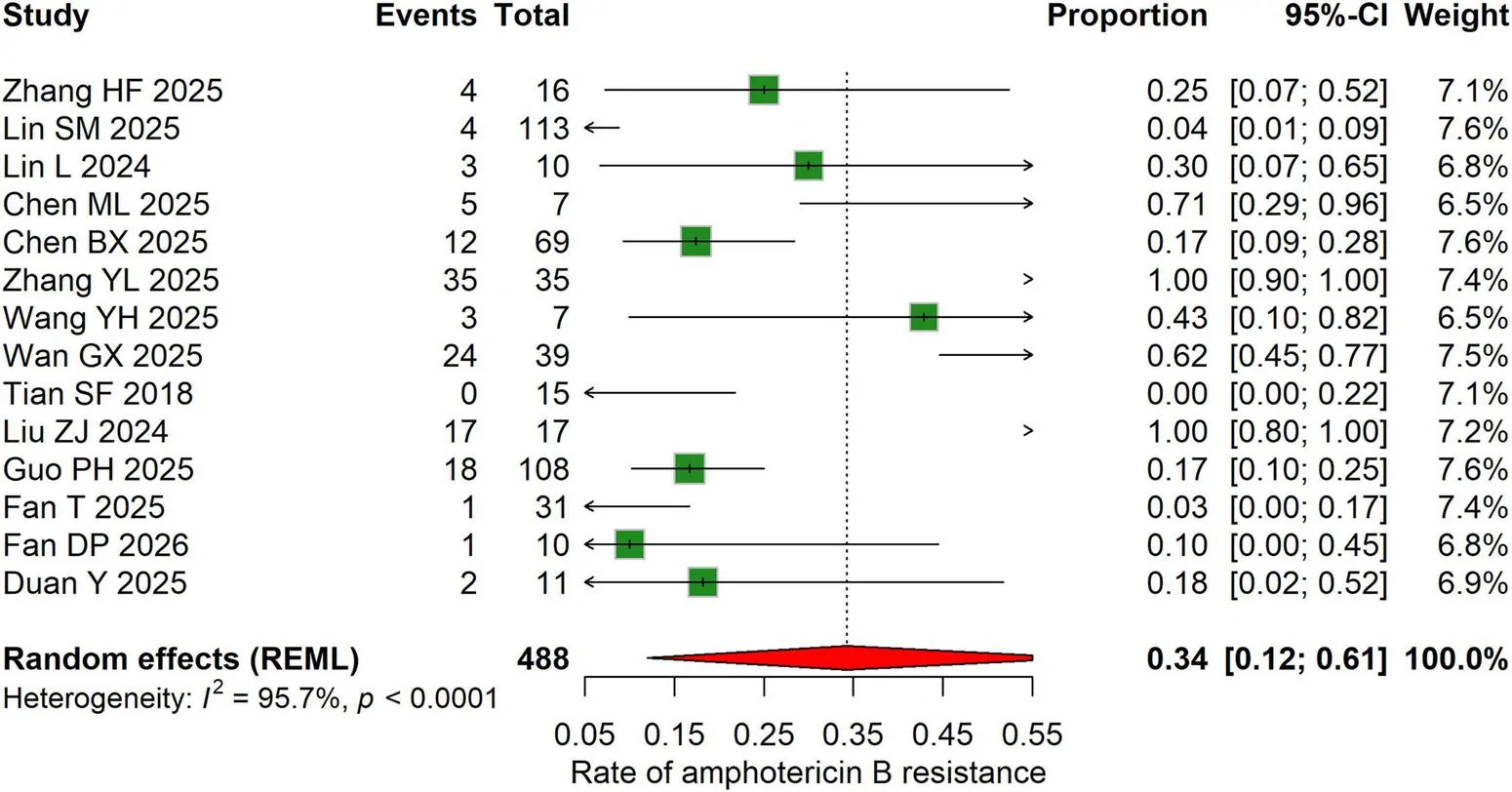
Forest plot of amphotericin B resistance rate in *C. auris* isolates.

A total of 488 strains were included for amphotericin B susceptibility evaluation, with 129 resistant strains. The random-effect model was used for pooled analysis, and the overall amphotericin B resistance rate was 34% (95% CI: 12%–61%), with statistically significant heterogeneity among included studies (*I*^2^ = 95.7%, *P* < 0.0001) (Figure 7).

### 3.4 Pooled multidrug resistance rate

**FIGURE 8:**
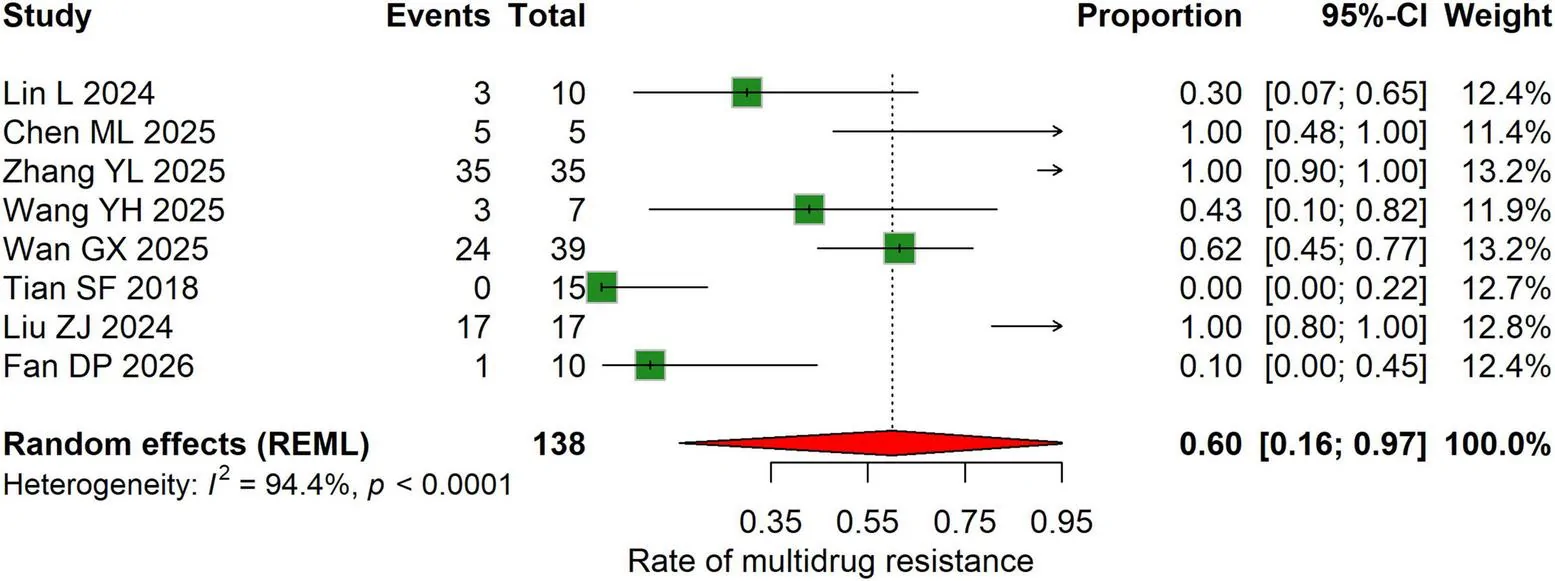
Forest plot of multidrug resistance rate in *C. auris* isolates.

Of the 138 isolates with complete susceptibility data for all three antifungal classes, 88 met the criteria for multidrug resistance, yielding a pooled MDR rate of 60% (95% CI: 16%–97%). Substantial heterogeneity was noted across studies (*I*^2^ = 94.4%, *P* < 0.0001) (Figure 8).

### 3.5 Meta-analysis results of poor prognosis indicators

**FIGURE 9:**
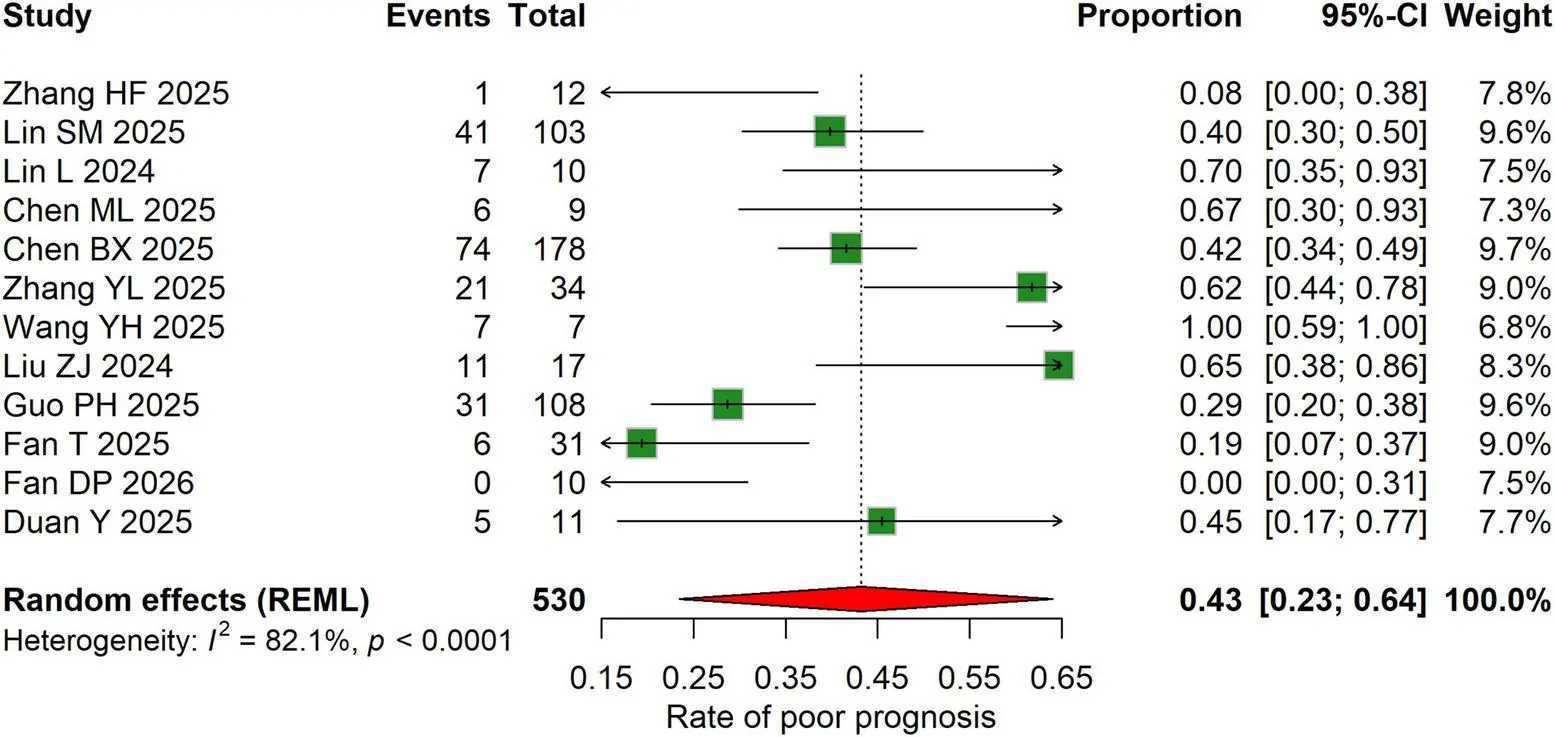
Forest plot of the poor prognosis rate among patients with *C. auris* colonization/infection.

Retrospective data on adverse prognosis before discharge were collected from 530 patients, among whom 210 had an adverse prognosis. Pooled analysis via the random-effect model showed that the overall incidence of adverse prognosis was 43% (95% CI: 23%–64%), with significant heterogeneity across studies (*I*^2^ = 82.1%, *P* < 0.0001) (Figure 9). Some studies reported stratified adverse prognosis, including in-hospital mortality rate and clinical deterioration rate at discharge. We further performed separate meta-analyses to estimate the pooled incidence of mortality and deterioration.

#### 3.5.1 Pooled in-hospital mortality rate

**FIGURE 10:**
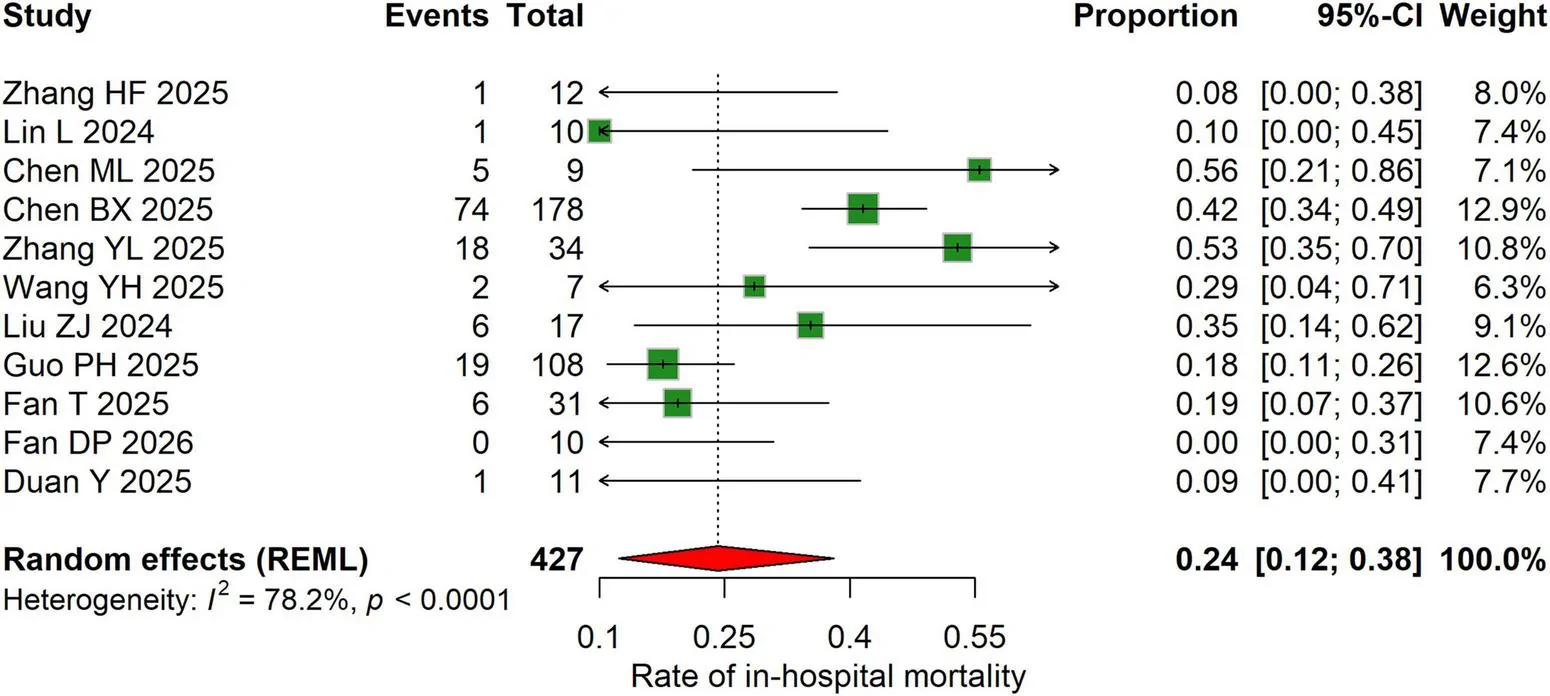
Forest plot of the in-hospital mortality rate among patients with *C. auris* colonization/infection.

We followed 427 patients with laboratory-detected *C. auris* isolates, 133 of whom sustained in-hospital mortality. Pooled analysis using the random-effect model showed that the in-hospital mortality rate was 24% (95% CI: 12%–38%), with statistically significant heterogeneity across studies (*I*^2^ = 78.2%, *P* < 0.0001) (Figure 10).

#### 3.5.2 Pooled clinical deterioration rate at discharge

**FIGURE 11:**
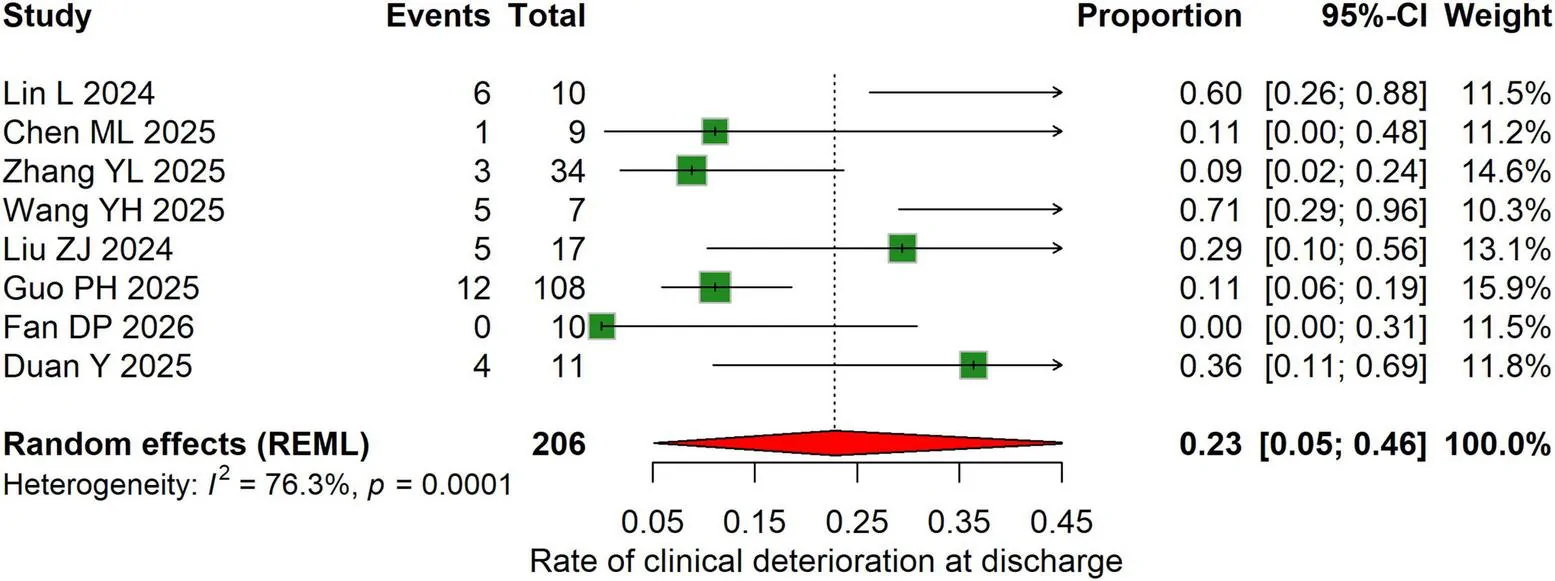
Forest plot of the clinical deterioration rate at discharge among patients with *C. auris* colonization/infection.

Clinical data of 206 patients positive for *C. auris* were analyzed, and clinical deterioration occurred in 36 patients at discharge. Pooled analysis using the random-effect model showed that the overall deterioration rate was 23% (95% CI: 5%–46%), with significant heterogeneity across studies (*I*^2^ = 76.3%, *P* = 0.0001) (Figure 11).

### 3.6 Subgroup analysis results of antifungal resistance rates

#### 3.6.1 Subgroup analysis of fluconazole resistance rate

Subgroup results showed that the pooled fluconazole resistance rate detected by the Sensititre YeastOne system was 100% (95% CI: 100%–100%), with no significant heterogeneity within the subgroup (*I*^2^ = 0%, *P* = 0.9712). There was a statistically significant difference in fluconazole resistance rates evaluated by different antifungal susceptibility testing methods (χ^2^ = 19.92, *P* < 0.0001) (Supplementary Figure 1). The pooled fluconazole resistance rate in northern China was 100% (95% CI: 100%–100%), with low heterogeneity within the subgroup (*I*^2^ = 0%, *P* = 0.9655). There was a statistically significant difference in fluconazole resistance rates between northern and southern China (χ^2^ = 3.37, *P* = 0.0663) (Supplementary Figure 2). In addition, the pooled fluconazole resistance rate of strains with *ERG11* gene mutation was 100% (95% CI: 100%–100%), with no statistically significant heterogeneity within the subgroup (*I*^2^ = 0%, *P* = 0.9403). Strains with the *ERG11* gene mutation had a higher fluconazole resistance rate than other isolated strains (χ^2^ = 8.25, *P* = 0.0041) (Supplementary Figure 3).

#### 3.6.2 Subgroup analysis of caspofungin resistance rate

For studies using the Sensititre YeastOne instrument, the pooled resistance rate for caspofungin was 5% (95% CI: 0%–14%), with substantial heterogeneity within this subgroup (*I*^2^ = 50.8%, *P* = 0.0708). There was no statistically significant difference in the caspofungin resistance rate across antifungal susceptibility testing platforms (χ^2^ = 2.95, *P* = 0.2284) (Supplementary Figure 4). The pooled caspofungin resistance rate in northern China was 7% (95% CI: 0%–39%), accompanied by moderate within-subgroup heterogeneity (*I*^2^ = 44.2%, *P* = 0.1667). There was no statistically significant difference in the caspofungin resistance rate between North and South China (χ^2^ = 0.54, *P* = 0.4636) (Supplementary Figure 5). Furthermore, the pooled caspofungin resistance rate among isolates found to carry *FKS1* mutations was 8% (95% CI: 3%–14%), with no significant heterogeneity within this subgroup (*I*^2^ = 0%, *P* = 0.5779). This rate was higher than that of other isolates (χ^2^ = 7.91, *P* = 0.0191) (Supplementary Figure 6).

#### 3.6.3 Subgroup analysis of amphotericin B resistance rate

Subgroup results showed that the pooled amphotericin B resistance rate detected by the Sensititre YeastOne system was 51% (95% CI: 2%–99%), with significant heterogeneity within the subgroup (*I*^2^ = 97.4%, *P* < 0.0001). There was a statistically significant difference in amphotericin B resistance rates evaluated by different antifungal susceptibility testing methods (χ^2^ = 11.89, *P* = 0.0026) (Supplementary Figure 7). The pooled amphotericin B resistance rate of *C. auris* in northern China was 74% (95% CI: 0%–100%), with significant heterogeneity within the subgroup (*I*^2^ = 96.6%, *P* < 0.0001). A marginally significant difference in amphotericin B resistance rates was observed between northern and southern China (χ^2^ = 2.74, *P* = 0.0976) (Supplementary Figure 8).

### 3.7 Subgroup analysis of multidrug resistance rate

In the subgroup analysis stratified by geographic region, the pooled MDR rate among isolates from northern China was 82% (95% CI: 0%–100%), with significant heterogeneity observed across studies within this subgroup (*I*^2^ = 96.6%, *P* < 0.0001). For southern China, the corresponding estimate was 37% (95% CI: 6%–76%), also with marked between-study heterogeneity (*I*^2^ = 71.3%, *p* = 0.0151). Although the MDR rate in the northern region appeared higher than that in the south, the difference between subgroups did not reach statistical significance (χ^2^ = 1.56, *P* = 0.2113) (Supplementary Figure 9).

### 3.8 Subgroup analysis results of poor prognosis indicators

#### 3.8.1 Subgroup analysis of in-hospital mortality rate

The in-hospital mortality rate among patients with laboratory-detected *C. auris* isolates in northern China was 48% (95% CI: 24%–73%), with no heterogeneity within the northern subgroup (*I*^2^ = 0%, *P* = 0.4641). The in-hospital mortality rate in southern China was 17% (95% CI: 7%–30%), with significant heterogeneity within the southern subgroup (*I*^2^ = 79.8%, *P* < 0.0001). The in-hospital mortality rate in northern China was significantly higher than that in southern China (χ^2^ = 14.86, *P* = 0.0001) (Supplementary Figure 10).

#### 3.8.2 Subgroup analysis of clinical deterioration rate at discharge

The clinical deterioration rate among patients with laboratory-detected *C. auris* isolates in northern China was 15% (95% CI: 0%–49%), with low heterogeneity within the subgroup (*I*^2^ = 38.5%, *P* = 0.1965). The clinical deterioration rate in southern China was 29% (95% CI: 0%–74%), with significant heterogeneity within the southern subgroup (*I*^2^ = 84.7%, *P* < 0.0001). There was no statistically significant difference in clinical deterioration rate between northern and southern China (χ^2^ = 0.88, *P* = 0.3471) (Supplementary Figure 11).

### 3.9 Publication bias analysis

**FIGURE 12:**
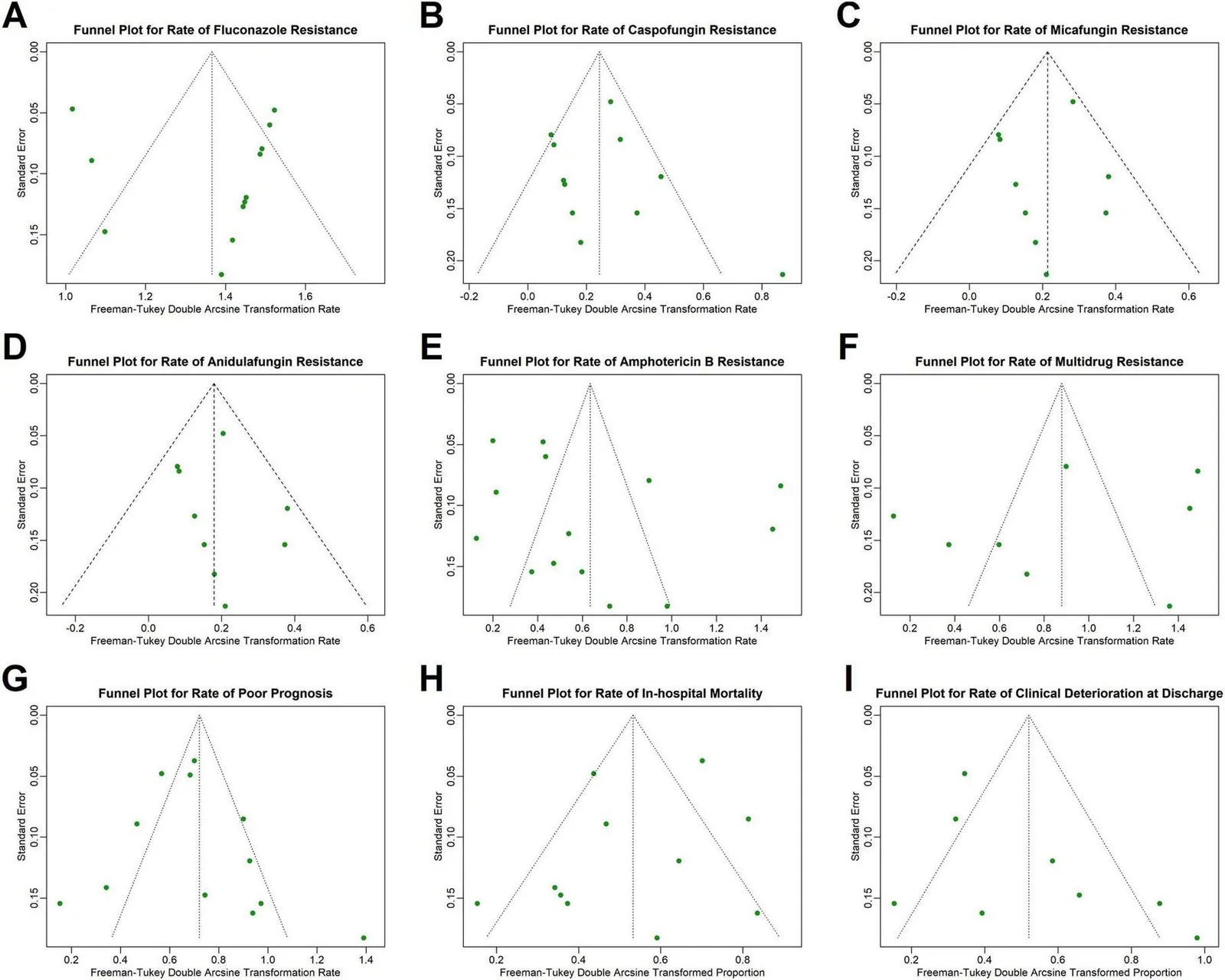
Funnel plots for publication bias assessment of pooled rates in *C. auris* isolates and patients. **(A)** Funnel plot for fluconazole resistance rate. **(B)** Funnel plot for caspofungin resistance rate. **(C)** Funnel plot for micafungin resistance rate. **(D)** Funnel plot for anidulafungin resistance rate. **(E)** Funnel plot for amphotericin B resistance rate. **(F)** Funnel plot for multidrug resistance rate. **(G)** Funnel plot for poor prognosis rate. **(H)** Funnel plot for in-hospital mortality rate. **(I)** Funnel plot for clinical deterioration rate at discharge.

Visual inspection of funnel plots for each outcome indicator showed varying degrees of asymmetry for fluconazole, caspofungin, micafungin, anidulafungin, amphotericin B, multidrug resistance, and clinical deterioration, whereas those for overall poor prognosis and in-hospital mortality were largely symmetrical. We further conducted Egger’s test for fluconazole, caspofungin, amphotericin B, overall poor prognosis, and in-hospital mortality. No significant publication bias was identified (*t* = 0.47, 0.46, 1.30, 0.66, -1.06; *P* = 0.6453, 0.6534, 0.2195, 0.5270, 0.3152, respectively) (Figure 12).

## 4 Discussion

Through systematic review and meta-analysis, this study comprehensively assessed the antifungal resistance rates and MDR rate of *C. auris* to fluconazole, caspofungin, micafungin, anidulafungin, and amphotericin B in mainland China, and also pooled the rates of adverse patient outcomes, including in-hospital mortality and clinical deterioration at discharge. In addition, subgroup analyses were conducted based on antifungal susceptibility testing methods, regional distribution, and *mutations in the ERG11 and FKS1 genes*, to explore patterns in the distribution of resistance rates to fluconazole, caspofungin, and amphotericin B as well as regional differences in MDR burden. Furthermore, we performed subgroup analyses to evaluate how mortality rates and clinical deterioration rates varied by region to better understand the epidemiological patterns. Overall, these findings provide important evidence-based medical references for choosing antifungals against *C. auris* and for preventing its spread in hospitals. They should also be useful for shaping future work in this field.

Our findings showed that the pooled resistance rate of *C. auris* to fluconazole in mainland China is 98% (95% CI: 93%–100%), reflecting an extremely high resistance burden. The heterogeneity of this pooled outcome was significant (*I*^2^ = 85.8%, *P* < 0.0001), and subgroup analysis revealed that differences in antifungal susceptibility testing methods may be one of the contributing factors. All *C. auris* tested using the Sensititre YeastOne method were resistant to fluconazole, with low heterogeneity (*I*^2^ = 0%), whereas studies using the ATB Fungus 3 method showed considerable variability. The resistance rates reported by Lin et al. (2024), Fan et al. (2025), and Duan et al. (2025) were 73% (95% CI: 64%–82%), 77% (95% CI: 59%–93%), and 82% (95% CI: 48%–99%), respectively. These rates contrasted sharply with the 100% (95% CI: 95%–100%) resistance rate reported by Chen and Feng (2025). This suggests that results may vary depending on the commercial antifungal susceptibility testing method used (Eraso et al., 2008; Zhang et al., 2014), which may contribute to the heterogeneity between studies. The resistance rate of *C. auris* to fluconazole in mainland China is similar to that of other Asian countries (Erdem et al., 2025; Thatchanamoorthy et al., 2026). Our subgroup analysis looked at geographic differences. For northern regions, all *C. auris* isolates in our study were resistant to fluconazole, giving a resistance rate of 100% (95% CI: 100%–100%), and the heterogeneity was low (*I*^2^ = 0%). The results from studies in the southern region were highly polarized. Some reported 100% resistance to fluconazole, while the resistance rates from Lin et al. (2025), Fan et al. (2025), and Duan et al. (2025) were relatively low. These variations mainly explain the prominent heterogeneity for southern isolates. From the analysis of the genetic sequences of the isolated strains, fluconazole resistance in *C. auris* is frequently linked to *ERG11* gene variants (Santana et al., 2026). The *ERG11* gene encodes a cytochrome P450 enzyme that participates in catalyzing the 14α-demethylation of lanosterol, a key limiting step in the synthesis of ergosterol in fungal cell membranes (Lepesheva et al., 2008). Fluconazole competes with this enzyme for binding, blocking the biosynthesis of ergosterol and thereby inhibiting the growth and proliferation of *C. auris*. In this study, *ERG11* gene missense mutations were detected in fluconazole-resistant *C. auris* from the South African Clade (Clade III) and the South Asian Clade (Clade I), including sites Y132F, V125A, F126L, and K143R. Such *ERG11* gene variants impair fluconazole’s suppression of fungal membrane biosynthesis and confer fluconazole resistance in *C. auris* (Liu F. et al., 2024; Wan et al., 2025; Wang et al., 2025; Zhang Y. et al., 2025). In contrast, none of the mutations were detected in fluconazole-sensitive strains, suggesting that the mutations at the *ERG11* gene sites in these strains are related to the difference in fluconazole resistance rates. Although the *ERG11* gene mutation was detected to be associated with fluconazole resistance, it must be pointed out that the resistance of *C. auris* is a complex phenotype resulting from the combined effect of multiple factors. The fluconazole resistance rate in this study is consistent with the 92.5% (95% CI: 89.5%–94.7%) reported globally (Achilonu et al., 2025). This finding indicates that fluconazole exhibits limited clinical efficacy against *C. auris* infections in mainland China and should no longer be administered as empirical treatment for suspected *C. auris* disease. This study constructed a funnel plot to assess publication bias by plotting the fluconazole resistance rates, and visually, the funnel plot showed a slight asymmetry, but the Egger’s test result did not indicate a significant publication bias (*P* = 0.6453). This minor asymmetry might be related to differences in study sample size and strain profiles.

In sharp contrast to fluconazole, the results of this meta-analysis show that the overall resistance rates of *C. auris* to the three echinocandin drugs in mainland China are relatively low. Echinocandins exhibit superior susceptibility against *C. auris* infections. Among them, the resistance rate of anidulafungin is the lowest, at 1% (95% CI: 0%–3%); micafungin follows, with a resistance rate of 2% (95% CI: 0%–4%); and caspofungin has a relatively higher resistance rate among the three, at 3% (95% CI: 0%–11%). Comparative analysis with global epidemiological data (Chen et al., 2020) reveals that the pooled caspofungin resistance rate in mainland China is significantly lower than the global average (12.1%). Meanwhile, the resistance rate of micafungin is much higher than the global average resistance rate (0.8%), and the resistance rate of anidulafungin was basically consistent with the global average of 1.1%. The overall resistance data suggest that echinocandins remain the preferred agents for treating *C. auris* infections in mainland China. However, we should be highly vigilant that the resistance of *C. auris* to caspofungin is showing a rapid upward trend globally, rising from 3.48% (Osei Sekyere, 2018) in 2017 to 12.1% (Chen et al., 2020) in 2019. This indicates that we should exercise caution when using this drug to prevent the resistance rate from rising further. Although the resistance level in mainland China is currently low, it is necessary to closely monitor the resistance trend to prevent the prevalence and spread of resistant strains. Analysis of genetic sequences of *C. auris* from mainland China shows that echinocandin-resistant strains carried mutations in the *FKS1* gene (Fan et al., 2026; Guo et al., 2025; Liu F. et al., 2024; Zhang Y. et al., 2025), with mutation sites including S639Y, S639F, W691L, F635I, R1354H, and W1471L. These mutations reduce the interaction between echinocandin drugs and fungal 1,3β-D-glucan synthase (Perlin, 2007; Wang and Xu, 2024), thereby hindering the drug’s ability to inhibit fungal cell wall synthesis. As a result, the drug fails to suppress *C. auris* proliferation, which ultimately enhances resistance. Mutant genes linked to antifungal resistance among Chinese *C. auris* isolates, as summarized in our analysis, matched those documented in international investigations (Chowdhary et al., 2023). These findings lend extra evidence that *FKS1* gene mutations correlate with echinocandin resistance in *C. auris*, and such genetic alterations partly account for the cross-study heterogeneity of caspofungin resistance rates. The funnel plot of caspofungin resistance rate drawn in this study is basically symmetrical, and the Egger’s test result indicates no significant publication bias (*P* = 0.6534), so the results of this study may be less affected by publication bias. Visually, the funnel plots of caspofungin and anidulafungin resistance rates are slightly asymmetric, with small-sample studies slightly concentrated at the bottom left. This finding is likely attributable to variations in sample size across the included studies and should not be interpreted as evidence of significant publication bias.

In mainland China, *C. auris* isolates exhibited a moderate level of resistance to amphotericin B, with a pooled resistance rate of 34% (95% CI: 12%–61%). The subgroup analysis of the susceptibility testing methods shows that there are significant differences in the resistance rates corresponding to different testing methods, suggesting that the testing methods are an important source of heterogeneity in the resistance rate. The ATB Fungus 3 method gave a significantly lower resistance rate than the Sensititre YeastOne method, which may be related to the principles and judgment criteria of the two methods. The ATB Fungus 3 method is a manual broth microdilution method, which has limited ability to detect low-level resistant strains and is prone to underestimating the resistance rate. In contrast, the Sensititre YeastOne method uses an automatic incubation and analysis system, which is more sensitive in detecting resistant isolates (Siopi et al., 2023), thus reporting a higher resistance rate. The subgroup analysis of the northern and southern regions in mainland China shows that the combined resistance rate in the northern region is 74% (95% CI: 0%–100%), and in the southern region it is 19% (95% CI: 8%–34%). The resistance rate in the northern region is slightly higher than that in the southern region (*P* = 0.0976), suggesting that regional distribution may be a factor influencing the amphotericin B resistance of *C. auris* in mainland China. The heterogeneity among the studies in the northern subgroup is at an extremely high level (*I*^2^ = 96.6%), and the stability of the results is poor. This phenomenon is mainly caused by an outlier study with a low resistance rate (Tian et al., 2018), whose resistance detection results are far lower than those of the other studies in the same subgroup (Chen et al., 2025; Liu F. et al., 2024; Zhang Y. et al., 2025). This difference causes a large span in the confidence interval of this subgroup, with a wide fluctuation range of the data. At the same time, the number of included studies in the northern region is small, and the total sample size is limited, resulting in insufficient test power and making it difficult to truly reflect the current resistance status in this region. The number of included studies in the southern region is sufficient, and the research data distribution is relatively concentrated, making the combined rates more reliable for reference. Variations in antifungal susceptibility testing protocols and regional characteristics aside, strain-specific genetic mutations constitute potential molecular drivers for inconsistent amphotericin B resistance rates across distinct studies. For instance, mutations in the *ERG6* gene that encodes sterol-methyltransferase (Rybak et al., 2022), alongside variants of transcription factor genes *RTG3* and *UPC2* (Chauhan et al., 2025), have been found to correlate with the occurrence of amphotericin B-resistant isolates. The resistance rate of *C. auris* to amphotericin B in this study is significantly lower than the global average resistance rate of 51.0% (95% CI: 42.3%–59.7%) (Achilonu et al., 2025), which may be related to regional differences in the genetic lineage of the strain, the level of clinical infection prevention and control, the frequency and exposure time of amphotericin B use, and other factors. We generated a funnel plot for the amphotericin B resistance rate. Visually, there were more points on the left side of the plot, but Egger’s test showed no significant publication bias (*P* = 0.2195). This inconsistency is probably due to several factors, including baseline heterogeneity and differences in sample sizes among the included studies.

This study included 138 isolates of *C. auris* that had undergone simultaneous susceptibility testing for fluconazole, echinocandin, and amphotericin B. The pooled MDR rate was 60%. The heterogeneity among the studies was extremely high (*I*^2^ = 94.4%), suggesting that the distribution of antifungal resistance phenotypes among the isolates varied among different studies. Multiple factors contributed to such heterogeneity. Mutations in the *ERG11* and *FKS1* genes, along with upregulation of efflux pumps, constitute the molecular basis for multidrug resistance in these isolates (Santana et al., 2026). The different genotypes of prevalent isolates in various regions further widen the differences in the results (Parnell et al., 2026). At the same time, the sampling modes could interfere with MDR rate calculation. Studies based on cluster outbreaks could obtain a large number of homologous MDR isolates at one time, and the proportion of antifungal resistance was significantly higher (Liu F. et al., 2024; Zhang Y. et al., 2025). Studies that only collected sporadic cases generally reported much lower rates of MDR (Fan et al., 2026; Tian et al., 2018). Moreover, the original studies failed to consistently distinguish between colonizing and infective isolates, making it difficult to eliminate the overall heterogeneity under multiple confounding factors. Standardized screening, contact isolation, and environmental disinfection can curb the spread of antifungal-resistant isolates within hospitals (Fan et al., 2026; Liu F. et al., 2024). Nevertheless, such infection control strategies can only reduce new infections and colonization caused by resistant isolates. They fail to directly lower the overall proportion of resistant isolates, exerting only an indirect influence on this indicator. The reported global MDR rate of *C. auris* ranges ranging from 41% to 58% (Achilonu et al., 2025; Lockhart et al., 2017), slightly lower than the pooled MDR rate of 60% in this study. This numerical difference need not necessarily stem from the biological characteristics of the isolates, but is largely due to biases arising from variations in study populations and sampling methods. After stratified analysis by northern and southern China, it can be observed that the pooled MDR rate was 82% in northern China, while it was only 37% in southern China. The Chi-square test showed no statistical difference between the two groups (*P* = 0.2113). Due to the limitations of the sample size in this study, it is currently impossible to confirm whether there are any real differences in MDR burden between regions. Therefore, MDR prevention and control for *C. auris* are equally vital in both northern and southern China. The funnel plot displayed asymmetric scatter distribution, and small-sample studies tended to report higher MDR rates, which indicated potential publication bias and might overestimate the overall pooled MDR rate. This asymmetric distribution may relate to great sample size variation and distinct baseline MDR rates of isolates from various regions. Great caution should be taken when interpreting the pooled MDR rate from this meta-analysis.

This study systematically analyzed the burden of adverse outcomes in patients with *C. auris* detected in mainland China. The overall rate of poor outcomes was as high as 43% (95% CI: 23%–64%), with an in-hospital mortality rate of 24% (95% CI: 12%–38%), which was lower than the global mortality rate of 39% (95% CI: 32%–47%) (Chen et al., 2020). Among them, the in-hospital mortality rate of patients in northern China was 48% (95% CI: 24%–73%), which was significantly higher than that in southern China [17% (95% CI: 7%–30%)]. Moreover, the heterogeneity within the northern region group was low (*I*^2^ = 0%, *P* = 0.4641), indicating relatively stable results. Variations in adverse clinical outcomes across regions may stem from clinical diagnosis and treatment levels, patients’ underlying disease characteristics, and strain prevalence differences (Erdem et al., 2025; Vaez et al., 2026). Multiple risk factors can increase the risk of *C. auris* infection in patients with compromised immune function (Lockhart et al., 2017; Nett, 2019), including chronic diseases such as hypertension and diabetes, concurrent pulmonary and urinary tract infections, and invasive medical procedures such as central venous catheterization and surgical operations. Patients in northern China may have more severe underlying medical conditions, resulting in higher in-hospital mortality. In addition, differences in the biological characteristics of strains can significantly affect clinical outcomes. Biofilm formation (Bing et al., 2023), accumulation of large lipid droplets (Zheng et al., 2025), and expression of the cell wall adhesion factor Scf1 (Santana et al., 2023) can enhance colonization and antifungal resistance, complicating efforts to clear fungal colonization and often correlating with unfavorable clinical outcomes. Contrary to the mortality rate, the clinical deterioration rate at discharge in southern China was 29% (95% CI: 0%–74%), higher than 15% (95% CI: 0%–49%) in northern China. Inter-study heterogeneity in southern China was high (*I*^2^ = 84.7%, *P* < 0.0001), which may be due to studies by Lin (Lin et al., 2024) and Wang (Wang et al., 2025). Most patients in these two studies suffered from sepsis or shock with prolonged hospitalization and a higher rate of clinical deterioration, widening the gap from other studies (Duan et al., 2025; Fan et al., 2026; Guo et al., 2025). Meanwhile, the warm, humid environment in southern China may facilitate prolonged colonization and survival of *C. auris* in hospital settings, on the skin, and on mucous membranes, potentially leading to recurrent infections and an increased risk of clinical deterioration at discharge. Visual inspection of the funnel plots for the overall poor prognosis rate and mortality rate showed that they were roughly symmetrical, and Egger’s test revealed no significant publication bias (*P* = 0.5270, 0.3152, respectively). This suggests that the pooled results for these two outcomes were relatively robust. Notably, cross-regional contrasts in in-hospital mortality and discharge deterioration rates require careful interpretation. Uneven counts of eligible studies, divergent patient baseline profiles, and varied hospital research backgrounds between northern and southern retrospective reports may create confounders that skew the apparent regional outcome gaps. The funnel plot for the clinical deterioration rate showed slight asymmetry, which was mainly attributed to the limited number of included studies and marked differences in sample size across studies.

Compared with prior meta-analyses, our study has several distinct advantages. Most earlier pooled analyses estimating antifungal resistance rates of *C. auris* centered on global or continental epidemiological profiles, with limited data specifically for mainland China. This review systematically integrated clinical and laboratory datasets obtained from healthcare facilities across mainland China, and the results are more suitable for clinical reference in China. Secondly, we systematically integrated and analyzed the epidemiological data on the resistance rates of the three commonly used classes of anti-*C. auris* drugs. We also innovatively summarized the adverse outcome indicators, such as patient mortality during hospitalization and deterioration of condition upon discharge. At the same time, we conducted subgroup analyses from three aspects: drug sensitivity testing methods, regional distribution, and key resistance genes. We also explored the sources of heterogeneity by combining the molecular resistance mechanisms of the strains. We systematically completed the integration analysis of the prevalence data of the resistance rate of *C. auris* and the adverse outcome indicators. Moreover, this study separately aggregated the resistance differences of caspofungin, micafungin, and anidulafungin in China, which can provide more detailed evidence-based guidance for individualized *C. auris* treatment in clinical practice.

This study has certain limitations: (1) All the studies were retrospective observational studies, and the included studies mainly focused on the eastern coastal regions, which may have introduced selection bias and affected the comprehensiveness of the data; (2) Some subgroup analyses included a small number of studies and limited sample sizes. For example, the subgroup analyses of amphotericin B resistance rate and multidrug resistance rate included studies with outlier estimates, resulting in high heterogeneity and poor stability of the pooled results. These findings require validation by future large-sample studies; (3) Original studies adopted different susceptibility testing devices and interpretive criteria. Although we performed subgroup analyses to explore this methodological heterogeneity, we could not fully explain all between-study variations. Resistance data derived from various testing platforms cannot be directly compared, and special caution should be taken when interpreting test results from the Sensititre YeastOne system; (4) Original studies did not fully report key confounding factors such as gender, age, underlying diseases, combined infections, invasive procedures, and prior antifungal exposure, and this study did not systematically correct the impact of these confounding factors on drug resistance and adverse prognosis; (5) Stratified data separating colonization and infection status were unavailable from original reports, so we pooled patients with *C. auris* colonization and infection for quantitative synthesis. *C. auris* isolates are frequently recovered from nonsterile sites, which usually indicate colonization rather than infection. Patients with simple colonization generally have a lower risk of adverse clinical events than those with infection. Combining these two distinct populations may distort pooled estimates of multidrug resistance prevalence and clinical outcome rates. Accordingly, our overall pooled results should be interpreted cautiously and cannot be generalized exclusively to patients with invasive *C. auris* infection; (6) We are unable to verify any direct causal relationship between *C. auris* isolation and negative clinical outcomes within this meta-analysis. A large proportion of included patients had severe chronic illnesses and concomitant infections, conditions widely recognized to independently raise mortality risks. These baseline comorbidities may substantially alter the observed association between *C. auris* colonization/infection and unfavorable clinical events. For this reason, our pooled data only show correlative patterns and cannot prove causal relationships; (7) The papers we pooled fell into two distinct categories: outbreak case series and standard retrospective clinical studies. Such variation in study design may distort pooled estimates of antifungal resistance and clinical outcomes. Furthermore, we could not fully rule out partial overlap of patient cohorts across different publications, which may bring additional bias to our synthetic results.

## 5 Conclusion

In short, *C. auris* in mainland China is highly resistant to fluconazole — so it should not be used empirically. Echinocandins remain largely effective and are first-line, though the rapid rise in caspofungin resistance worldwide needs close attention. Amphotericin B only exerts moderate activity against this pathogen and requires careful risk-benefit evaluation in clinical practice. High rates of multidrug resistance are prevalent nationwide, which calls for long-term dynamic surveillance of resistance shifts. Regionally, northern patients face higher in-hospital mortality, while southern patients carry a greater risk of clinical deterioration by discharge. Further research is needed to fill existing knowledge gaps, limit nosocomial transmission of *C. auris*, and slow the development of antifungal resistance. Nationwide standardized multicenter resistance surveillance covering central and western regions should be promoted to balance geographically uneven data distribution, and unified antifungal susceptibility testing criteria should be established to reduce methodological heterogeneity. Multi-omics analyses will facilitate exploration of multidrug resistance regulatory pathways beyond single-gene mutations. Additionally, prospective cohort studies differentiating colonization from infection should be carried out to validate the pooled effect sizes of this meta-analysis. Furthermore, hospitals should refine environmental decolonization protocols and sustain long-term surveillance of caspofungin resistance trends to stop broad dissemination of resistant isolates.

## Footnotes

1 https://www.crd.york.ac.uk/PROSPERO/view/CRD420261363661.

2 https://www.cdc.gov/candida-auris/hcp/laboratories/antifungal-susceptibility-testing.htm.

## Supplementary material

Supplementary Figure 1 Subgroup analysis of the rate of fluconazole resistance in *C. auris* isolates, stratified by antifungal susceptibility testing method.

Supplementary Figure 2 Subgroup analysis of the rate of fluconazole resistance in *C. auris* isolates, stratified by geographic region (Northern vs. Southern China).

Supplementary Figure 3 Subgroup analysis of the rate of fluconazole resistance in *C. auris* isolates, stratified by *ERG11* mutation status.

Supplementary Figure 4 Subgroup analysis of the rate of caspofungin resistance in *C. auris* isolates, stratified by antifungal susceptibility testing method.

Supplementary Figure 5 Subgroup analysis of the rate of caspofungin resistance in *C. auris* isolates, stratified by geographic region (Northern vs. Southern China).

Supplementary Figure 6 Subgroup analysis of the rate of caspofungin resistance in *C. auris* isolates, stratified by *FKS1* mutation status.

Supplementary Figure 7 Subgroup analysis of the rate of amphotericin B resistance in *C. auris* isolates, stratified by antifungal susceptibility testing method.

Supplementary Figure 8 Subgroup analysis of the rate of amphotericin B resistance in *C. auris* isolates, stratified by geographic region (Northern vs. Southern China).

Supplementary Figure 9 Subgroup analysis of the rate of multidrug resistance among patients with *C. auris* colonization/infection, stratified by geographic region (Northern vs. Southern China).

Supplementary Figure 10 Subgroup analysis of the rate of in-hospital mortality among patients with *C. auris* colonization/infection, stratified by geographic region (Northern vs. Southern China).

Supplementary Figure 11 Subgroup analysis of the rate of clinical deterioration at discharge among patients with *C. auris* colonization/infection, stratified by geographic region (Northern vs. Southern China).
